# Late presentation to HIV/AIDS care at the Douala general hospital, Cameroon: its associated factors, and consequences

**DOI:** 10.1186/s12879-018-3204-8

**Published:** 2018-07-03

**Authors:** Henry Namme Luma, Paulia Jua, Olivier-Tresor Donfack, Felicite Kamdem, Eveline Ngouadjeu, Hugo Bertrand Mbatchou, Marie-Solange Doualla, Yakouba Njankou Mapoure

**Affiliations:** 1 Douala General Hospital, P.0. Box 4856, Douala, Cameroon; 2 Faculty of Medicine and Biomedical Sciences, University of Yaoundé 1, Yaoundé, Cameroon; 3 Faculty of Health Sciences, University of Buea, Buea, Cameroon; 4 Faculty of Medicine and Pharmaceutical Sciences, University of Douala, Douala, Cameroon

**Keywords:** HIV/AIDS, Late presentation, Associated factors, Mortality, Opportunistic infections, Cameroon

## Abstract

**Background:**

The introduction of anti-retroviral treatment (ART) has significantly reduced mortality and morbidity associated with HIV/AIDS. While treatment at early stages of the disease is related to a better prognosis, late presentation (LP) to care is harmful to the infected person, the society and is more costly. We aimed to describe late presentation to HIV care, its associated factors and consequences in patients followed up in a tertiary hospital in Cameroon.

**Methods:**

We retrospectively assessed patients’ files between 1996 and 2014 at the Douala general hospital (DGH) HIV treatment centre. Late presentation (LP) to HIV care was defined as a CD4+ T cell count< 350 cells/mm3 or advanced clinical stages of the disease (WHO stages 3/4) at first presentation for care. We used logistic regression to study factors associated with late presentation and assessed occurrence of opportunistic infections and mortality at 3, 6 and 12 months after presentation to care.

**Results:**

Of 1866 files studied, mean age was 40 (SD: 10) years, median CD4+ T cell count was 147 (IQR: 63–270) cells/mm3, 58.2% were at HIV clinical stages 3 and 4. The prevalence of late presentation to HIV care was 89.7% (95% CI: 88.2–91.0%) and remained above 80% from 1996 to 2014. Circumstances of diagnosis: prevention of mother to child transmission program/blood donation (OR = 0.16, 95% CI 0.10–0.29), having a positive partner (OR = 0.16, 95%CI = 0.10–0.26), and routine screening (OR = 0.13, 95%CI = 0.10–0.19) reduced the odds of presenting late compared to clinical suspicion. Students had lower odds of presenting late compared to people who had an employment (OR = 0.50, 95%CI = 0.26–0.98). Calendar time OR = 1.64, 95% CI = 1.08–2.48 for ≥2010 vs. < 2005) increased the odds of late presentation. Mortality and opportunistic infections prevalence remained significantly higher in late presenters at 3, 6 and 12 months than in early presenters.

**Conclusion:**

Late presentation to HIV care is very high at the DGH and is related to poor outcome. More screening and sensitization campaigns should be carried out in the population to diagnose the disease at an earlier stage.

## Background

Globally, there are approximately 36.7 million people living with HIV, of whom about 70% are in Africa [1]. The burden associated with HIV infection has decreased over the past decade as access to anti-retroviral treatment (ART) has increased. In 2015, WHO recommended that all people living with HIV start ART irrespective of clinical or immune status and Cameroon national guidelines adopted this recommendation in 2016. Despite this change, people living with HIV continue to present to care late and with advanced disease [2]. During the natural course of HIV infection, there is a progressive loss of CD4+ T cells [3–5] and, this leads to a severely immuno-compromised state in the infected host. Unfortunately, in many countries, a substantial number of HIV infected individuals still do not enter health care until late in their infection course [5].

Different criteria have been used to define late presentation (LP). These generally include CD4 cell count and/or AIDS defining disease [6–8]. However a consensus definition has been validated [9]. Late presentation was defined as persons presenting for care with a CD4+ T cell count below 350 cells/mm3 or presenting with an AIDS-defining event, regardless of the CD4 cell count. Advanced Late presentation is presentation for care with a CD4+ T cell count below 200 cells/mm^3^, or presenting with an AIDS-defining event, regardless of the CD4+ T cell count [9]. LP remains a significant problem worldwide involving both high and low income countries. Reported prevalence in Europe range from 15 and 66% [10, 11] in Asia, 72–83% [5] and in Africa 35–89% [12–16]. Factors associated with LP have been shown to include age, male sex, low level of education, single status, rural residency, fear of stigmas, ignorance and fear of perceived adverse effects of ART [17–20].

LP is associated with significantly heightened levels of HIV related morbidity and mortality [21], increased risk of untoward transmission and high health care costs [22–24]. Surveillance to identify the extent to which LP occurs is crucial and remains inadequate [9]. There is very limited published data in sub-Saharan Africa (SSA), and Cameroon in particular, to the best of our knowledge. In studying the prevalence of LP, we will better comprehend the magnitude of those at increased risk of clinical disease progression. This will also be useful to improve surveillance and assist health professionals in allocation of resources for improved HIV care, especially with the current “test and treat” campaigns in Cameroon.

We therefore aimed to describe late presentation to HIV disease care and to identify its associated factors in a large referral hospital treatment centre between 1996 and 2014.

## Methodology

### Study design and setting

A retrospective analysis of clinical files of HIV/AIDs patients attending the DGH accredited treatment centre (ATC) from January 1996 to December 2014 was carried out.

The DGH is a tertiary health institution situated in Douala, the economic capital of Cameroon which has a catchment population of about 3 million inhabitants. It is home to one of the centres of excellence in HIV/AIDS management in the region providing active care to about 2000 HIV infected individuals. The centre employs a multidisciplinary team approach to HIV/AIDS management consisting of specialists in infectious diseases, internal medicine, neurology, chest medicine, as well as psychologists, pharmacists, primary care physicians, nurses and social workers.

The majority of the patients seen are from the Douala locality. However, some patients with difficult management issues (complications of AIDS) are temporarily referred to this center for improved management. Diagnosis, follow up and treatment of patients are carried out according to national guidelines developed by the National AIDS Control Committee for use in all HIV treatment centres in Cameroon.

Once a patient is diagnosed HIV positive in any setting, the individual is referred to the ATC for counseling. Under the supervision of the attending physician, a medical file is opened. Socio-demographic and clinical data of that patient are obtained and a pre-therapeutic workup is then requested. At weekly meetings, files of new patients (as well as old patients whose treatment needs to be modified) are presented and there are deliberations on the most appropriate management strategy. In accordance with the National Aids Control Committee guidelines, patients with CD4+ T cell counts ˂ 500cells/mm3 were commenced on ART. This cut off was upgraded in accordance with the WHO guidelines of 2013. The initial cut offs were < 200cells/mm3 from 2006 to 2010, <350cells/mm3 from 2010 to 2013, <500cells/mm3 from 2013 to 2015, and “treatment for all” in 2016 (74–78). The hospital pharmacy dispenses ART and some prophylactic drugs as indicated, for opportunistic infections at no cost to the patients. Patient’s prescriptions are renewed monthly following a physicians’ visit.

Every 6 months, CD4+ T cell count, full blood count, liver transaminases and blood urea/creatinine are requested with results recorded in the files. Viral Load measurements are also recommended yearly though most patients cannot afford it.

### Participants

Included in this study were; files of patients aged 18 and above, registered and followed up at the DGH ATC for at least 1 year following presentation to care, with at least two clinic visits, and having recorded relevant initial diagnostic information (clinical stage at presentation and/or initial CD4 counts at HIV diagnosis). Excluded were files of patients that were incomplete (no initial diagnostic information) and files of those who had commenced care in another centre. As the DGH plays a supervisory role on other smaller hospitals and health centres in the region, severely ill cases referred for better management and referred back after improvement were also excluded from this study.

### Data collection

We consecutively included exploitable files. Incomplete files were excluded at the level of data collection and not at the level of the analysis. A sensitivity analysis could therefore not be done. A structured, pretested data collection form was used to obtain information from patients’ files. Information collected were; socio-demographic (age, sex, occupational status - employed, unemployed or student, religion (Christians, Muslims and others), marital status - single, married, divorced or widowed, nationality (nationals and non-nationals) and residence (urban or rural). Clinical information included; WHO clinical stage of HIV at presentation for care, circumstance of diagnosis (Prevention of mother to child transmission program/blood donation, HIV positive partner, routine screening) and presence of any opportunistic infections at time of diagnosis. Laboratory data were; CD4+ T cell counts, viral load, transaminases (alanine aminotransferases – ALT and aspartate aminotransferases - AST), blood urea/creatinine results at HIV diagnosis. The same laboratory investigations and their results were monitored throughout the first and second years following diagnosis. Any opportunistic infections occurring within the 2 year period were also noted. (See Table 4 in Appendix for data collection tool).

### Definition of terms

Presentation at HIV care was categorized as early or late presenters. Late presentation was defined as persons presenting for care with a CD4+ T cell count below (<) 350cells/mm^3^ or WHO stages 3 or 4 regardless of the CD4+ T cell count. Late presenters with advanced disease were those who presented for care with a CD4+ T cell count <200cells/mm^3^ or WHO stages 3 or 4 regardless of the CD4 T cell count [9]. Early Presenters were individuals presenting for care with CD4+ T cell counts >350cells/mm^3^ or at WHO stage 1 or 2.

Circumstances of diagnosis included:

1. Prevention of mother to child transmission program (PMTCT): patients tested for HIV through pre-natal screening.
2. Clinical suspicion: HIV testing was requested by the patient or health personnel as a result of ill health.
3. Routine screening: Patient requested the HIV test without any apparent illness, or HIV test requested prior to a medical intervention.
4. Partner is positive: where the patient’s sexual partner(s) is/are diagnosed positive prompting their own testing.
5. Blood donation: where the patient is diagnosed following routine screening for blood donation.

Predictor variables were CD_4_ count and WHO stage at presentation to care. Outcome variables were; presence of Opportunistic infections, immune reconstitution and death within 1 year from presentation to HIV care.

For patients lost to follow up (no health visits or clinic attendance for at least 6 months), disease evolution and mortality was assessed through phone calls to either the patients themselves or their next of kin after informed verbal consent. These phone numbers were derived from their files.

Ethical clearance for the study was sought and obtained from the institutional review board IRB) of the Faculty of Medicine and Pharmaceutical Sciences (Reference number IEC-UD/488/02/2016/T) and administrative clearance from the DGH permitting use of patient files.

### Statistical analysis

The outcome of this study was late presentation to HIV diagnosis at the Douala general hospital, and was defined as having a CD4+ T cell count done at least within 3 months of diagnosis < 350 cells/mm3 or being at an advanced stage of the disease (WHO stage 3 or 4) at diagnosis. Results were presented as count (percent), and the chi2 test was used to examine the association between variables. For analytical purposes, we further categorised variables using clinical cut-offs and regrouped others with no a priori categorisation or clinical cut-off values (age, circumstances of diagnosis, marital status, and calendar year). The Wald test was used for trends. To assess factors associated to late presentation at diagnosis, we used univariate logistic regression reporting Odds Ratios (OR) and their 95% Confidence intervals (CIs). We finally used all factors that showed at least a weak evidence of association (*p* value < 0.10) with the outcome alongside age, to build an exploratory multivariate logistic regression model. Results were analysed using STATA 13, and the threshold for significance was set at the level of 5%.

## Results

Of the 3904 files studied, 1710 were incomplete and 194 had less than a year follow up. There were thus 2000 eligible files of which 134 did not have either the initial CD4 cell count and/or the WHO clinical stage (impossibility to define late presentation using our criteria). We finally included 1866 files in our analysis.

Baseline characteristics: Out of the 1866 files, 55% belonged to males, mean age was 40 (SD: 10) years; median initial CD4+ T cell count was 147 (IQR: 63–270) cells/mm3. Three hundred and eighty-four (20.5%) had CD4+ T cell counts below 50 cells/mm3, 804 (43.1) were between 50 and 199 cells/mm3, 392 (21.2%) between 200 and 349 cells/mm3, and 282 (15.1%) from 350 cells/mm3 above; while 58.2% (1083/1860) were at WHO stages 3 and 4 of the disease (Table 1). Of participant files with information on the period of first CD4+ T cell count, 89.4% were done within 3 months from diagnosis while only 1.7% were done after 3 months. On presentation to care, 77.4% were started on Cotrimoxazole, while 23.4% initiated ARV (Table 1).

**Table 1:** Baseline characteristics of the study population

Characteristics	N (%)
Mean age, *N* = 1866	40(±10)
Sex, *N* = 1866	
Male	1026 (55.0)
Female	840 (45.0)
Religion, *N* = 1851	
Christian	1744 (94.2)
Muslim	107 (5.8)
Calendar Year, *N* = 1866	
< 2005	494 (26.5)
2005–2009	717 (38.4)
≥ 2010	655 (35.1)
Cotrimoxazole, *N* = 1861	
No	420 (22.6)
Yes	1441 (77.4)
ARV, *N* = 1861	
No	1426 (76.6)
Yes	435 (23.4)
WHO stage, *N* = 1860	
Stage1	297 (15.9)
Stage2	480 (25.8)
Stage3	598 (32.2)
Stage4	485 (26.0)
Marital status, *N* = 1866	
Single	643 (34.5)
Married	1012 (54.2)
Divorced + Widowed	211 (11.3)
Employment, *N* = 1866	
Employed	1271 (68.1)
Unemployed	522 (27.9)
Student	73 (4.0)
Circumstances of diagnosis, *N* = 1865	
Routine screening	311 (16.7)
PMTCT program	81 (4.3)
Blood donation	8 (0.4)
HIV positive partner	119 (6.4)
Clinical suspicion	1346 (72.2)
CD4+ T cell count, *N* = 1866	
< 50 cells/mm3	384 (20.6)
50–199 cells/mm3	804 (43.1)
200–349 cells/mm3	392 (21.2)
≥ 350 cells/mm3	282 (15.1)
Time of first CD4+ T cell test, *N* = 1858	
The day of diagnosis	166 (8.9)
Within 3 months	1661 (89.4)
> 3 months	31 (1.7)

Results are presented as count (percentage) or otherwise stated. ARV and Cotrimoxazole started at presentation

*PMTCT* Prevention of mother to child transmission program

**Fig. 1:**
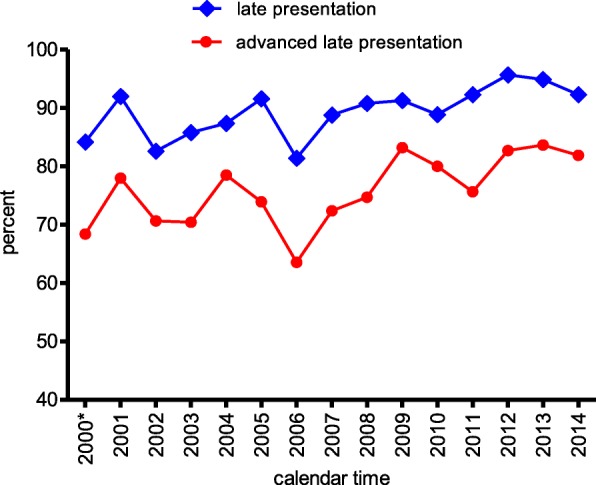
Prevalence of late presentation over calendar time. 2000*: we regrouped 1996 to 2000 because of sparsity of data

Prevalence of late presentation and trend: The overall prevalence of late presentation was 89.7% (1672/1866), 95% CI: 88.2–91.0% and that of Advanced late presentation was 1425/1866 = 76.4% (95% CI: 74.4–78.2%). Figure 1 depicts the prevalence of late presentation and advanced late presentation over calendar time. Late presentation remained very high (above 80%) over the years.

Risk factors of late presentation: in univariate analysis, the odds for late presentation was increased by calendar time (OR = 1.29, 95% CI = 0.91–1.83 for 2005 to 2009 and OR = 1.99, 95% CI = 1.34–2.95 for ≥2010vs. < 2005). Students had lower odds of presenting late compared to people who had an employment (OR = 0.37, 95%CI = 0.21–0.67). Patients who were diagnosed through PMTCT program or blood donation (OR = 0.16, 95%CI: 0.10–0.28), those who were diagnosed because they had a positive partner (OR = 0.15, 95%CI = 0.10–0.25), and those who were diagnosed by routine screening (OR = 0.12, 95%CI = 0.10–0.18) had reduced odds of presenting late compared to those who were diagnosed because of clinical suspicion (Table 2). After adjusting factors by each other and by age in multivariate logistic regression, people who were diagnosed after 2010 had an increased odds of presenting late compared to those who were diagnosed before 2005 (OR = 1.64, 95%CI = 1.08–2.48), inversely, student had 50% lower odds (OR = 0.50, 95%CI = 0.26–0.98) of presenting late compared to employed people. People who were diagnosed via routine screening, PMTCT or blood donation or because of a positive partner had respectively 82, 82 and 87% lower odds of presenting late compared to those who were diagnosed because of clinical suspicion (Table 2).

**Table 2:** Factors associated to late presentation

Factors	Total	Early presentation	Late presentation	OR (95%CI)	*P* value	aOR (95% CI)	*P* value
Age, tertiles							
< 35	688	80 (11.6)	608 (88.4)	Ref		Ref	
35–43	577	52 (9.0)	525 (91.0)	1.33 (0.92–1.92)	0.31	1.05 (0.70–1.59)	0.46
≥ 43	600	61 (10.2)	539 (89.8)	1.16 (0.82–1.65)		0.82 (0.55–1.23)	
Sex							
Women	840	82 (9.7)	758 (90.3)	Ref			
Men	1026	112 (10.9)	914 (89.1)	0.87 (0.64–1.18)	0.37		
Calendar Year							
< 2005	494	68 (13.6)	426 (86.4)	Ref		Ref	
2005–2009	717	78 (10.9)	639 (89.1)	1.29 (0.91–1.83)	0.002	1.12 (0.77–1.62)	0.05
≥ 2010	655	48 (7.3)	607 (92.7)	1.99 (1.34–2.95)		1.64 (1.08–2.48)	
Marital status							
Married	1012	111 (10.9)	910 (89.1)	Ref			
Single	643	56 (8.6)	587 (91.4)	1.31 (0.94–1.85)	0.14		
Divorced + Widowed	211	27 (12.8)	184 (87.2)	0.84 (0.54–1.32)			
Employment status							
Employed	1271	121 (9.5)	1150 (90.5)	Ref		Ref	
Unemployed	522	57 (10.9)	465 (89.1)	0.85 (0.61–1.19)	0.003	0.98 (0.69–1.40)	
Student	73	16 (21.9)	57 (78.1)	0.37 (0.21–0.67)		0.50 (0.26–0.98)	0.12
Religion							
Christian	1744	185 (10.6)	1559 (89.4)	Ref			
Muslim	107	9 (8.4)	98 (91.6)	1.29 (0.64–2.59)	0.48		
Circumstances of diagnosis							
Clinical suspicion	1346	60 (4.5)	1286 (95.6)	Ref		Ref	
PMTCT + Blood donation	89	20 (22.5)	69 (77.5)	0.16 (0.10–0.28)		0.16 (0.10–0.29)	
HIV positive partner	119	28 (23.5)	91 (76.5)	0.15 (0.10–0.25)	< 0.0001	0.16 (0.10–0.26)	< 0.0001
Routine screening	311	86 (27.4)	225 (72.6)	0.12 (0.10–0.18)		0.13 (0.10–0.19)	

*aOR* Adjusted odds ratio, *PMTCT* Prevention of mother to child transmission

**Fig. 2:**
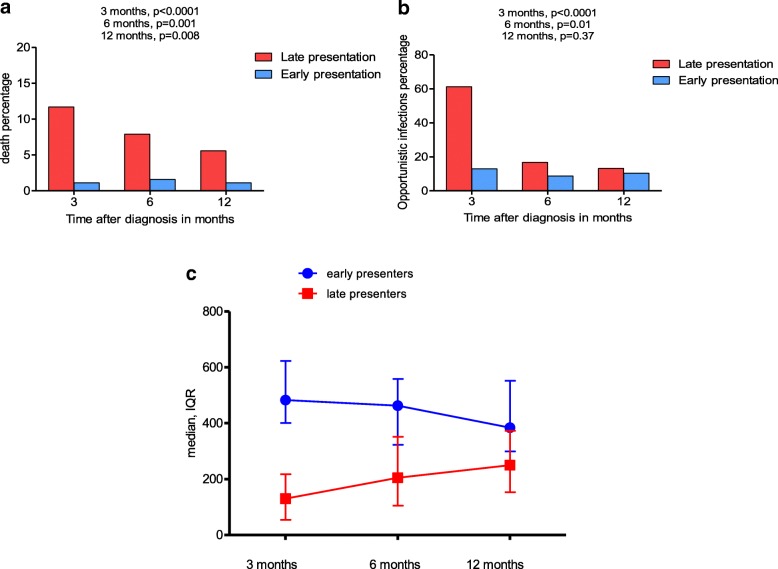
**a** Mortality at 3, 6 and 12 months after presentation to care. **b** Opportunistic infections at 3, 6 and 12 months after presentation to care. **c** Progression of CD4+ T cell counts at 3, 6 and 12 months after presentation to care

Consequences of late presentation: There were significantly more deaths in late presenters than in early presenters at 3 months (184/1575 = 11.6% vs. 2/189 = 1.1%), 6 months (117/1470 = 7.9% vs. 3/190 = 1.6%) and 12 months (76/1356 = 5.6% vs. 2/187 = 1.1%) respectively (Fig. 2a). The same pattern of death was observed amongst the subgroup that initiated ARV (Table 4 in Appendix). Similarly to deaths, OIs were more frequent in late presenters compared to early presenters at 3 months (1024/1670 = 61.3% vs. 25/192 = 13.02%), 6 months (202/1201 = 16.8% vs. 13/149 = 8.7%), and 12 months (131/991 = 13.2% vs. 14/134 = 10.4%) (Fig. 2b) and the same trends were observed in the subgroup that initiated Cotrimoxazole prophylaxis (Table 4 in Appendix). There was a significant rise (*p* < 0.0001) in CD4 cell counts in the first 12 months in late presenters. At 12 months median CD4+ cell counts were still lower than in early presenters. In early presenters, there was a slight decrease in the trend of CD4+ T cell counts at 6 and 12 months which was not statistically significant (*p* = 0.284) (Fig. 2c). The most common OI was pulmonary tuberculosis with respective values of 20.8% (116/1044), 30.1% (64/213), and 25% (36/144) at 3, 6 and 12 months after diagnosis (Table 3).

**Table 3:** Common opportunistic infections in the study population at 3, 6 and 12 months after presentation to care

Infections	3 months		6 months		12 months	
	n/N	%	n/N	%	n/N	%
Pulmonary tuberculosis	218/1046	20.8	64/213	30.1	36/144	25.0
Bacterial pneumonia	116/1044	11.1	25/213	11.7	23/143	16.1
Herpes zoster	169/1041	16.2	26/213	12.2	14/143	9.8
Skin Kaposi sarcoma	98/1042	9.4	16/213	7.5	13/143	9.1
Candidiasis	41/1045	3.9	11/213	5.2	13/143	9.1
Cryptococcal Meningo-encephalitis	49/1047	4.7	10/215	4.7	5/144	3.5
Cerebral toxoplasmosis	85/1051	8.1	10/213	4.7	11/144	7.6
Tuberculous meningitis	25/1047	2.4	8/213	3.8	6/137	4.2
HIV encephalitis	27/1047	2.6	1/213	0.50	7/143	4.9
Pulmonary aspergillosis	1/1044	0.10	0/213	0.00	0/143	0.00
Pulmonary Kaposi sarcoma	12/1044	1.2	13/213	6.1	9/143	6.3
Microsporidia	1/1043	0.10	1/213	0.50	1/143	0.70
Isosporidia	3/1043	0.30	2/213	1.0	2/143	1.4
Cryptosporidiosis	2/1043	0.20	0/213	0.00	2/143	1.40

## Discussion

We aimed to describe late presentation, and its associated factors, to HIV care at the DGH between 1996 and 2014. The prevalence of late presentation to HIV care was 89.7% and remained above 80% during the study period. Circumstances of diagnosis (PMTCT program/blood donation, HIV positive partner, routine screening) and employment (student) categories reduced the odds of presenting late, whereas calendar time (2010 above) and diagnosis following clinical suspicion increased the odds of late presentation. Mortality and opportunistic infection (mostly prominently, pulmonary tuberculosis) prevalence remained significantly higher in late presenters at 3, 6 and 12 months.

Following a global consensus, ambitious goals were set for the year 2020, known as the 90–90–90 target [25]. This called for 90% of people living with HIV knowing their HIV status, 90% of people who know their status receiving treatment and 90% of people on HIV treatment having a suppressed viral load [25]. In spite of this, many people still remain unaware of their HIV status and cascade for care for HIV varies widely from country to country and WHO region to region [5].

Knowledge of the prevalence of late presentation can be used as an essential indicator for monitoring prevention programs, and also in assessing the effectiveness of testing campaigns [26]. With set targets, introduction and improvement in ART uptake, the prevalence of late presentation to HIV care did not seem to decrease over time worldwide [2] .

In this study, using the consensus definition for LP, we found a prevalence of almost 90%. This is very similar to findings in a study in neighbouring Nigeria [16]. Our findings were however very high when compared to most other studies in SSA. The criteria for LP were different and thus the importance of using a consensus definition. For example, an Ethiopian study [20] using the criteria of CD4 < 200 found LP to be 61.8%. This is comparable to our own findings if the same definition was used (63.7%). Other authors in SSA found a lower proportion of LP compared to ours as in Malawi 75.4% [27] and Uganda 59.2% [28]. In most of the SSA studies, the sample size was smaller and fewer patients had complete files with data such as CD4 count and clinical stage on entry compared to this study. In European studies, the prevalence of LP using the consensus definition ranged between 50 and 70% [21, 29, 30]. We also found out in this study that the prevalence of LP remained consistently high over the study period. In a Nigerian study, there was a significant decrease in the proportion of individuals presenting late over a 5 year period. However, as in this study LP was 80% and above [16]. This was contrary to a Cambodian study where LP decreased significantly over time from 67% in 2003 to 41% in 2013. These results suggest that in the absence of targeted HIV prevention and diagnostic campaigns, the proportion of HIV patients with LP will continue to be a major problem. Patients will be quite immune suppressed at presentation and as in this study, justifying the high proportion of patients commenced on Cotrimoxazole prophylaxis (77.4%). Cotrimoxazole prophylaxis for toxoplasmosis and pneumocystis in Cameroon is commenced at CD4+ cell counts below 200 cells/mm3.

Several socio-economic factors have previously been shown to be associated with LP [17–20]. Unfortunately we could not evaluate most of them in this study. However, our student population had significantly lower odds of presenting with LP compared to other populations (Employed and unemployed). Similarly those who were employed were less likely to present late than those who were unemployed as found by another author [15]. This finding may be linked to the fact that young people in Cameroon aged 15 to 24 were shown to have better knowledge on HIV/AIDS [25].

Patients who were diagnosed because of clinical suspicion of HIV infection, had higher odds of LP. Having an HIV test due to illness was also found to be an independent risk factor associated with LP to HIV/AIDS care in Harare and Ethiopia [17, 31]. This could mean that individuals attended testing centres primarily when they developed AIDS related conditions. The Public Health message derived from this finding is that LP might be a direct consequence of late diagnosis of HIV and ultimately late linkage to care and treatment [31]. Patients commonly tested during prenatal care, blood donation, diagnosis of a positive partner or routine screening, were less likely to present late. They are therefore more likely linked to care with a higher CD4+ T cell count compared to individuals tested only upon showing symptoms of HIV. The high prevalence of LP provides additional evidence to shift towards routine testing and linkage to care rather than risk based strategies that might not effectively or efficiently engage individuals infected with HIV [32].

We clearly demonstrated that there were increased numbers of OIs (with Tuberculosis as the leading cause) and mortality in late than in early presenters. The management of patients presenting late is challenging. Some of them may die because of AIDS defining illness even before having the possibility to start ART, and individuals with very low CD4+ T cell count have a higher probability of developing complications on starting ART [30]. Unfortunately, a substantial proportion of people who are late presenters are still dying from the disease [33].

Our results should be treated with caution particularly, with regards to reproducibility in different HIV settings. It could be argued that the tertiary referral setting may receive patients with more severe disease and thus a higher prevalence of LP. However other authors have found similar results [16, 34]. The possible drop in CD4+ T cell counts during infections may lead to overestimates of LP when the consensus definition is applied [26]. Also, when based on the consensus definition, LP may be apparently overestimated, nonetheless it can be used as evidence to reinforce current guidelines advocating “test and treat” regardless of CD4+ T cell counts. Other limitations include: some inconsistencies in the data; most especially, misclassification bias and measurement errors (reliability) as we assessed files retrospectively over calendar time; missing data as variables were extracted from clinical files which gave us very little flexibility in our analyses and limited the amount of variables that could be investigated (such as associated factors and also follow up over a longer period of time than 12 months.) Nevertheless, the strength of this study is related to the large number of patients included and the high quality of the data presented in this setting with availability of CD4+ T cell counts and HIV clinical stage in over 1800 patient files on presentation to care.

## Conclusion

Nine out of ten HIV/AIDS patients attending the DGH ATC are late presenters to HIV care. This has grave consequences to the individual. Targeted public health interventions to improve early entry into HIV care are urgently needed if Cameroon is to attain the 90–90-90 objective for 2020.

## Appendix

**Table 4:** Death in ARV treated and OIs in patients on Cotrimoxazole prophylaxis

Death	3 months		6 months		12 months	
Initiated ARV	n/N	%	n/N	%	n/N	%
Early presenters	1/29	3.5	1/30	3.3	1/29	3.5
Late presenters	124/1304	9.5	105/1254	8.4	67/1154	5.8
Opportunistic infections	3 months		6 months		12 months	
Initiated Cotrimoxazole	n/N	%	n/N	%	n/N	%
Early presenters	6/21	28.6	4/19	21.1	3/16	18.8
Late presenters	913/1418	64.4	176/1002	17.6	116/818	14.2

## Abbreviations

AIDS: Acquired Immune Deficiency Syndrome
AOR: Adjusted odds ratio
ART: Antiretroviral therapy
ATC: Accredited treatment Centre
CI: Confidence interval
DGH: Douala General Hospital
HIV: Human Immunodeficiency Virus
IQR: Interquartile range
OR: Odds ratio
SSA: Sub-Saharan Africa
WHO: World Health Organization
